# Measuring COVID-19 related stress and its associated factors among the parents of school-aged children during the first lockdown in France

**DOI:** 10.1186/s12889-023-16731-3

**Published:** 2023-09-19

**Authors:** Stéphanie Bourion-Bédès, Hélène Rousseau, Martine Batt, Carole Beltrand, Rabah Machane, Pascale Tarquinio, Cyril Tarquinio, Cédric Baumann

**Affiliations:** 1 Centre Hospitalier de Versailles, Service Universitaire de Psychiatrie de L’Enfant Et de L’Adolescent, 78000 Versailles-Le Chesnay, France; 2 Université Paris-Saclay, UVSQ, Inserm U1018, CESP, ‘DevPsy’, 94807 Villejuif, France; 3 Methodology, Data Management and Statistics Unit, University Hospital of Nancy, 54000 Nancy, France; 4 InterPsy, GRC Team, University of Lorraine, 54000 Nancy, France; 5 Pierre Janet Center, University of Lorraine, 57 000 Metz, France; 6 UR 4360 APEMAC (Health Adjustment, Measurement and Assessment, Interdisciplinary Approaches), University of Lorraine, 54000 Nancy, France

**Keywords:** COVID-19, Stress, Parents, Mental health, Resilience, Family support

## Abstract

**Background:**

The COVID-19 pandemic placed important challenges on parents, as they had to meet various demands during lockdown, including childcare, work and homeschooling. Therefore, the current study aimed to investigate perceived stress levels among the parents of school-aged children and explore their association with sociodemographic, environmental and psychological factors during lockdown.

**Methods:**

A cross-sectional study was conducted among the parents of school-aged children ages 8 to 18, who lived in the Grand Est region of France during the first wave of the pandemic. An online survey collected sociodemographic data, living and working conditions, and exposure to COVID-19 as well as parent’s levels of perceived stress (PSS-10), self-perceived health status (SF-12), social support (MSPSS) and resilience (BRS). Multivariable logistic regression models were conducted to evaluate the association between moderate to severe perceived stress and various factors.

**Results:**

In total, 734 parents were included. The results indicated that 47% were experiencing moderate stress and 7.2% were experiencing severe stress. Factors most strongly associated with risk of moderate to severe levels of stress were lower levels of parental resilience (OR = 3.8, 95% CI: 2.2–6.6) and poor self-perceived mental health status (OR = 7.3, 95% CI: 5.0–10.8). The following risk factors were also identified: female sex; being in the age range of 35–44; difficulties isolating and contracting COVID-19, which involved hospitalization and separation or isolation from family. The support of friends (OR = 0.8, 95% CI: 0.7–1.0) and family (OR = 0.5, 95% CI: 0.3–0.8) were protective factors.

**Conclusions:**

These findings suggest that supportive and preventive programs should focus on the improvement of resilience and mental health management to promote parents’ wellbeing. Research has to focus both on individuals’ inner potential for increasing resilience and the environmental resources to be activated. Building and boosting resilience among parents could serve as a protective factor against negative outcomes for them and their families.

## Background

In December 2019, coronavirus disease 2019 (COVID-19) was first identified in Wuhan, China [1]. Since then, it spread rapidly, affecting other parts of China and many other countries worldwide [2]. The World Health Organization (WHO) declared the coronavirus epidemic a pandemic on March 11, 2020, [3] and stated on March 13 that Europe had become the new epicenter of the pandemic [4]. This prompted governments to implement disease containment measures, such as school closures, home lockdowns, social distancing and travel restrictions, that disrupted daily routines and changed family life. Children experienced a prolonged state of physical isolation from their extended families, their peers and their teachers [5], and many parents were forced to work remotely in addition to homeschooling their children.

A widely used definition of a stressful situation is one in which the demands of a situation threaten to exceed the resources of the individual [6]. Several studies have found that high stress was a common initial reaction to this pandemic [7, 8], but if stressors are too persistent and too strong, then they may have mental health effects such as anxiety, depression and reduced quality of life [9].

Among stressors during the pandemic, those involving uncertainty surrounding the pandemic, fear for one’s own health, fear of infecting others, reduced social and physical contact with others, poor information from public health authorities, potential financial loss and numerous unexpected challenges for many families have resulted in important psychosocial consequences for parents [10]. A previous work on the topic showed that parents in the United States experienced higher levels of stress during COVID-19 than adults without children, with nearly half of the parents of children under the age of 18 (46%) reporting a high stress level [11]. Another study revealed that parental stress increased substantially during COVID-19 and has not yet returned to pre-COVID-19 levels [10], highlighting the need for resources and support to manage parents’ stress. A cross-sectional survey of adults living in Canada identified that parents of children under 18 who lived at home during the first wave were at a disproportionate risk of worsened mental health due to the COVID-19 pandemic, with a larger proportion of parents under 18 reporting increased alcohol consumption and suicidal thoughts or feelings than the rest of the population. Thus, 44.3% of parents of children < 18 living at home reported worse mental health as a result of COVID-19 pandemic compared with 35.6% of respondents without children < 18 living at home [12]. Moreover, rising levels of parental stress place children at risk of neglect, violence, or abuse [13]. In consideration of the needs of diverse families, several organizations, such as the WHO, UNICEF and the Centers for Disease Control and Prevention (CDC), have collaborated to provide online, open access parenting resources during COVID-19 [14].

Research on resilience is important because it has been found to be negatively related to distress and positively related to mental health [15]. Although there is no consensus on its definition, the American Psychology Association describes resilience as the process of adapting well or “bouncing back” in the face of, trauma, tragedy, threats, adversity or significant sources of stress [16]. Previous studies have highlighted the key role of resilience in adapting to disasters or pandemics as individuals were more likely to suffer adverse mental and psychological consequences when they were not equipped with sufficient levels of resilience and coping ability [17, 18]. Indeed, parents’ resilience has been suggested to be a crucial protective factor for psychological adjustment to COVID-19 [19]. Thus, this study aims to 1) assess perceived stress among parents and 2) investigate how sociodemographic characteristics, living and working conditions, family relations, social support, parents’ self-perceived health status and resilience are related to perceived stress in the study sample. We hypothesized that moderate to high perceived stress would be significantly associated with poor resilience. As resilience is a modifiable factor and stressful events associated with COVID-19 may reoccur, examining its role could aid in the design and implementation of public health strategies to improve resilience as a tool for protection against adversity.

## Methods

### Participants and procedures

The data analyzed were from the observational Feelings and Psychological Impact of the COVID-19 Epidemic among Children and Adolescents in the Grand Est area (PIMS2-CoV19) study. This was a cross-sectional survey that was conducted from May 26 to July 6, 2020 while this geographical area was under a partial lockdown, a period during which people no longer needed an attestation to move within 62 miles of home, but parks, gardens, middle and high schools remained closed. The Grand Est region incurred a high incidence of COVID-19, with 19.6 cases per 100,000 inhabitants during the survey period. It was one of the three most affected regions in France. From a complete list including 408 middle and high schools in the Academy of Metz-Nancy, 46 schools were randomly selected using proportionate stratification for the baseline school identification and recruitment. In selected establishments, the online survey was disseminated through institutional mailing lists to parents of school children aged 8 to 18. The principals’ dissemination of the online survey to parents was unknown. However the number of parental connections to the survey link was identified, such as the number of parents who provided parental agreement to participate and completed the questionnaires related to the study. The survey took approximately 20 min to complete. Participation was voluntary, and informed consent was obtained from all participants. The study protocol was approved by the Commissioner for Data Protection (Comité National Informatique et Liberté-registration 2220408), in accordance with the principles of the Declaration of Helsinki.

### Measures

#### Demographic information

Parents completed an ad hoc 26-item self-reported demographic information questionnaire including age, sex, marital status, highest education level, employment status (i.e., not working or working full time/part time) prior to the epidemic, current work situation during lockdown and home location. Information about children’s age and the time spent by parents helping with their children’s homework were also collected but the information on the name of school that a child went to was not available. Parents were also asked about their living conditions, the presence of a relative or acquaintance infected with COVID-19 and whether there were family conflicts during the pandemic or noises inside or outside the residence.

#### Stress 

The 10-item Perceived Stress Scale (PSS-10) is a useful self-report stress scale that was derived from an original 14-item form [20]. It includes 10 items that reflect the frequency of an indicator of stress over the past month. Items were rated on a 5-point scale ranging from “never” (= 0) to “very often” (= 4) and summed. Total scores range from 0 to 40, with higher scores indicating higher levels of perceived stress. Based on previous cutoff values, total scores are categorized as follow: low stress (0–13), moderate stress (14–26) and high stress (27–40) [21]. The French version of the PSS-10 has previously shown good internal consistency and reliability [22].

#### Self-perceived health status

Participants completed the generic French SF-12 instrument to assess their self-perceived health status [23]. Its twelve items provide a global health status regarding both physical and mental health. A mental health component summary (MCS) and a physical health component summary (PCS) are calculated using the 12 items. Both scores range between 0 and 100, with a higher score indicating better self-perceived health status.

#### Resilience

The Brief Resilience Scale (BRS) was used to assess the ability to recover from stress [24]. It consists of six items, among which items 2, 4 and 6 use negative wording. Participants responded to each item using a 5-point rating scale for which 1 indicates “very strongly disagree” and 5 indicates “very strongly agree”. Items 2, 4 and 6 were reversed scored, and the overall BRS score was the mean of the six items, with a higher score indicating greater resilience. Scores ranging from 3 to 4.3 indicated a normal level of resilience. A score < 3 was indicative of low resilience, whereas a score > 4.3 indicated high resilience [25]. The French version of the scale showed satisfactory psychometric properties [26].

#### Self-perceived social support

The Multidimensional Scale of Perceived Social Support (MSPSS) has evolved to be one of the scales most widely used to assess self-perceived social support. Its twelve items measure social support in three domains: family (items 3, 4, 8 and 11), friends (items 6, 7, 9 and 12), and significant others (items 1, 2, 5 and 10) [27]. Respondents are asked to rate their responses on a 7-point Likert scale ranging from 1 (“very strongly disagree”) to 7 (“very strongly agree”). The total item scores are summed for each subscale following the instructions used for scoring the MSPSS; the score of each subscale ranges from 1 to 7, with higher scores indicating higher perceived social support. The French version of the scale is reported to possess adequate psychometric properties [28].

### Statistical analyses

All analyses were performed using SAS 9.4 (SAS Inst., Cary, NC, USA). Frequencies and proportions were used for categorical variables. Continuous variables were described by the mean and standard deviation or the median, as appropriate. First, descriptive analysis was conducted to describe the sociodemographic characteristics, living conditions and mental health status of the respondents. Second, a logistic regression analysis was performed to explore significant associations between the sample characteristics (sociodemographic characteristics, living and working conditions, family conflicts, time spent on children’s homework, concerns regarding the health threat posed by COVID-19, parents’ self-perceived health status, resilience and self-perceived social support scores) and a moderate to high level of stress during the COVID-19 partial lockdown. The PCS and MCS were analyzed in binary form, using the median to split the sample, and resilience was also analyzed in binary form because of the recommended cutoff value for low resilience of < 3 [25]. Relevant factors were identified as those found to be associated in the bivariate analysis at the 5% threshold and were retained for multivariable analyses. Estimates of the strength of associations were demonstrated by odds ratios (ORs) with a 95% confidence interval (CI). Statistical significance was assumed for *p* < 0.05. The goodness of fit was assessed by calculating the model determination coefficient (R2) and the percentage predicted by the model. The lack of correlation and multicollinearity was verified. The Hosmer and Lemeshow test was used to compare and select the best multivariable model.

## Results

### Sociodemographic, working and living condition characteristics

We counted 2763 parental connections to the survey link, and 1742 parents provided parental agreement to participate. In total, complete data were obtained for 734 parents. The data on sociodemographic, working and living conditions are depicted in Tables 1 and 2. Parent respondents consisted of 633 females (86.2%) and 101 males (13.8%) aged between 35 to 45 years (51.5%). In terms of family status, 74.4% were married or living with the other parent. The majority were employed before the COVID-19 outbreak, with 63.3% participants having full time work and 30.4% having had their work interrupted due to the COVID-19 outbreak. Most participants lived in rural areas (61.1%), and 5.3% reported having no access to outdoor areas. More than one-sixth of the parents (18.4%) stated that they have had conflicts with family members, and 15.0% reported difficulties isolating at home. Among the sample, 8.3% of the parents stated that they had experienced loud noise outside the residence, and 4.8% reported loud noises inside the home. Regarding homeschooling, two-fifths of the parents (42.6%) reported spending 2 h or less per day helping their children. Nearly one-third of the respondents (29.8%) reported that someone at home, or a relative or acquaintance, had been infected with COVID-19.

**Table 1:** Sociodemographic and working characteristics of the parents’ sample population under the lockdown (*N* = 734)

	**Full sample**	
	N	%
**Characteristic**		
**Gender**		
Male	101	13.8
Female	633	86.2
**Age**	734	
< 35	67	9.1
35–45	378	51.5
> 45	289	39.4
**Marital status (missing = 3)**		
Married/live with the other parent	544	74.4
Separated/divorced/widowed	164	22.5
Single parent	23	3.1
**Parental education level (missing = 1)**		
Less than high school	279	38.1
Higher education	454	61.9
**Occupational status before the lockdown (missing = 11)**		
Looking for employment	60	8**.**3
Full-time work	458	63.3
Part-time work	168	23.2
Retired/ student	37	5. 1
**Occupational status during the lockdown (missing = 31)**		
Work interruption	214	30.4
Telecommuting full-time worker at home	257	36.6
Full-time worker at work	155	22.0
Most time worker at work, time remaining at home	34	4.8
Most time worker at home, time remaining at work	43	6.1
**Time spent on children schoolwork at home (missing = **2**)**		
< 2 h a day	312	42.6
2–4 h a day	266	36.3
≥ 4 h a day	154	21.0

**Table 2:** Living conditions of the parents’ sample population under lockdown (*N* = 734)

	**Full sample**	
	*N* = 734	
	N	%
**Living arrangement**s** (missing = 3)**		
Alone with children	115	15.7
With the other parent and children	544	74.4
With a partner other than the parent and children	72	9.8
**Number of individual**s** confined at home**		
< 4	221	30.1
4	334	45.5
> 4	179	24.4
**Home location (missing = 9)**		
Urban area	282	38.9
Rural area	443	61.1
**Access to a private outside space (missing = 1)**		
No access	39	5.3
Private balcony, courtyard or terrace	82	11.2
Private domestic garden	586	79.9
Courtyard or garden for collective use	26	3.5
**Frequency of exiting the house during the lockdown (missing = 1)**		
Several times a day	108	14.7
Once a day	174	23.7
Several times a week	124	16.9
Once a week	130	17.7
Less than once a week	98	13.4
Never leaving home	99	13.5
**Noises outside the residence**		
Yes	61	8.3
No	673	91.7
**Noises inside the residence**		
Yes	35	4.8
No	699	95.2
**Difficulty isolating at home**		
Yes	110	15.0
No	624	85.0
**Tensions and conflicts at home**		
Yes	135	18.4
No	599	81.6
**Someone at home/relative or acquaintance had COVID-19**		
None	426	58.0
Confirmed and hospitalized cases	66	9.0
Confirmed and non-hospitalized cases	153	20.8
Suspected cases	89	12.1

### Parental levels of stress, self-perceived health status, social support and resilience

Table 3 shows the results obtained from the scales. Of the 734 parents, 398 reported experiencing stress (54.2%), among whom stress levels were moderate in 345 (47.0%) and severe in 53 (7.2%).

**Table 3:** Parental stress, social support and resilience scores under the lockdown

	**Full sample**	
	*N* = 734	
	N	%/Mean (SD)
**PSS-10 total score**		
Low stress (0–13)	326	45.8
Moderate stress (14–26)	345	47.0
High stress (27–40)	53	7.2
**SF-12 PCS score**	734	69.6 (13.0)
**SF-12 MCS score**	734	55.3 (16.7)
**MSPSS-total score**	734	5.5 (1.2)
**MSPSS-subscales**		
Family	734	5.5 (1.3)
Friend	734	5.3 (1.4)
Significant other	734	5.7 (1.3)
**BRS-total score**	698	
BRS < 3	155	22.2
BRS ≥ 3	543	77.8

*Abbreviation*: *SD* Standard deviation, *PSS-10* Perceived Stress Scale-10, *MSPSS* Multidimensional Scale of Perceived Social Support, *BRS* Brief Resilience Scale

The mean SF-12 scores were 69.6 (SD = 13.0) (median 74.2, interquartile range 64.6–77.8) and 55.3 (SD = 16.7) (median 58.4, interquartile range 42.4–69.2) for the PCS and MCS domains, respectively. The mean MSPSS total score was 5.5 (SD = 1.2). The mean scores for support from family, friends and significant others were 5.5 (SD = 1.3), 5.3 (SD = 1.4) and 5.7 (SD = 1.3), respectively. Of the 734 parents, only 698 completed the BRS scale, and among them, one-fifth (22.2%) presented a low level of resilience (BRS < 3).

### Factors associated with moderate to severe parental stress

The results of the bivariate and multivariable analyses of the factors associated with stress are presented in Table 4. In the bivariate analysis, variables were identified as significant when *p* < 0.05, which indicates that the OR value of one variable was statistically significant. A model was computed with a stepwise selection of candidate variables that used an entry level of significance of 0.05 and a stay level of significance of 0.05. The model determination coefficient (R^2^) was 0.35 and the Hosmer and Lemeshow Goodness-of-Fit test was 0.50. Among sociodemographics, female sex (OR = 2.5, 95% CI: 1.4–4.4) and parents between 35–44 years of age (OR = 2.4, 95% CI: 1.2–4.9) were more likely to have moderate to high levels of stress. Among environmental factors, parents who reported difficulties isolating at home (OR = 2.2, 95% CI: 1.2–3.9) and who had confirmed COVID-19 cases among their immediate or extended families, regardless of whether these confirmed cases involved hospitalization (OR = 2.4, 95% CI: 1.2–4.8) or did not involve hospitalization (OR = 1.8, 95% CI: 1.1–2.9), were associated with a moderate to severe level of stress. In terms of psychological factors, parents with high levels of support from their families (OR = 0.5, 95% CI: 0.3–0.8) and friends (OR = 0.8, 95% CI: 0.7–1.0) were less likely to have moderate to severe levels of stress. Those with a low level of resilience (OR = 3.8, 95% CI: 2.2–6.6) and a poor self-perceived health status (OR = 7.3, 95% CI: 5.0–10.8) were more likely to score above the cutoff for moderate stress.

**Table 4:** Factors associated with parental moderate to severe stress level during COVID-19 lockdown

	**Bivariate regression analysis**			**Multivariable logistic regression analysis** (*N* = 698) R^2^ = 0.35, H&L = 0.50		
	**OR**	**95% CI**	***P-value***	**OR**	**95% CI**	***P-value***
**Age** (ref: < 35)			0.041			0.031
35–44	1.4	0.8–2.3		2.4	1.2–4.9	
≥ 45	0.9	0.6–1.6		1.7	0.8–3.6	
**Gender** (ref: male)	3.0	1.9–4.6	< 0.0001	2.5	1.4–4.4	0.002
**Living arrangements (ref:** with the other parent or partner and children**)**			0.323			
Alone with children	1.2	0.8–1.8				
**Parental education level** (ref: higher studies**)**			0.530			
Less than high school/ High school graduate	0. 9	0.7–1.2				
**Time spent on children’s schoolwork at home** ( ref: < 4 h a day**)**			0.123			
≥ 4 h a day	1.3	0.9–1.9				
**Home location** (ref: urban vs rural area)	1.0	0.7–1.3	0.750			
**Occupational status during the lockdown** (ref: work interruption)			0. 214			
Telecommuting full-time worker at home	0.8	0.6–1.2				
Full-time worker at work	1.1	0.7–1.7				
Most time worker at work, time remaining at home	1.5	0.7–3.2				
Most time worker at home, time remaining at work	0.6	0.3–1.1				
**Number of individuals confined at home (ref:** > 4)			0.946			
< 4	0.9	0.6–1.4				
4	0.9	0.7–1.4				
**Access to a private outside space (ref: no acce**ss)			0. 635			
Private balcony, courtyard or terrace	0.7	0.3–1.4				
Courtyard or garden for collective use	1.0	0.4–2.8				
Private domestic garden	0.7	0.4-.1.4				
**Difficulty isolating at home** (Yes vs No)	3.8	2.3–6.2	< 0.0001	2.2	1.2–3.9	0.012
**Tensions and conflicts at home** (Yes vs No)	4.5	2.8–7.1	< 0.0001			
**Noises outside the housing** (Yes vs No)	2.1	1.2–3.8	0.009			
**Noises inside the housing** (Yes vs No)	2.2	1.0–4.6	0.041			
**Someone at home/ relative or acquaintance had COVID-19** (ref: no)			0.014			0.027
Confirmed and hospitalized cases	2.1	1.2–3.7		2.4	1.2–4.8	
Confirmed and non-hospitalized cases	1.5	1.1–2.2		1.8	1.1–2.9	
Suspected cases	1.1	0.7–1.7		1.1	0.6–1.9	
**MSPSS subscales**						
Family (ref: ≤ median MSPSS score)	0.2	0.2–0.3	< 0.0001	0.5	0.3–0.8	0.002
Friend	0.7	0.6–0.8	< 0.0001	0.8	0.7–1.0	0.023
Significant other (ref: ≤ median MSPSS score)	0.4	0.3–0.5	< 0.0001			
**PCS SF-12 score** (ref: ≥ median PCS score)	1.7	1. 2- 2.2	0.0007			
**MCS SF-12 score** (ref: ≥ median MCS score)	11.3	8.0- 16.0	< 0.0001	7.3	5.0–10.8	< 0.0001
**BRS-total score** (ref: score ≥ 3)						
BRS < 3	7.1	4.4–11.4	< 0.0001	3.8	2.2–6.6	< 0.0001

*Abbreviations*: *OR* Odds ratio, the probability of PSS-10 score > 13; OR < 1, decreased frequency of PSS-10 score > 13; OR > 1, increased frequency of PSS-10 score > 13; *SD* Standard deviation, *MSPSS* Multidimensional Scale of Perceived Social Support, *BRS* Brief Resilience Scale

## Discussion

Our findings highlight that approximately half of the French parents living in the Grand Est Area experienced moderate psychological stress, and approximately 7% experienced severe psychological stress. The prevalence of moderate and high stress at the time of the study suggests that the epidemic placed a mental health burden as of May 26, 2020, with more than 149,000 confirmed cases and over 28,500 deaths attributable to COVID-19 reported in France. These results are consistent with previous findings among the Chinese general population, with nearly half of the sample having moderate psychological stress and 4.5% having severe psychological stress [29]. However, the rates of moderate and severe stress in our study were below the rates among US parents with > 1 child who completed an online survey in May 2020 and among whom one-in-five reporting high levels of stress and nearly 70% reporting moderate levels of stress [10]. These differences in the prevalence of perceived stress may be due to health, cultural, economic and policy differences between countries that may affect public health differently during the pandemic [30].

This study focused on the factors that influence the stress level of parents. Examining sociodemographic variables as predictors of moderate to high levels of stress yielded some interesting findings. For example, female sex was found to be a significant risk factor, which agrees with past research that has shown that women are more susceptible to stress than men and that they tend to experience greater emotional responses [31]. This is consistent with the current literature that indicates gender differences in the psychological responses to COVID-19 [32] when closures of schools, playgrounds and community facilities increase the challenge for women of juggling work, homeschooling and childcare all by themselves. Previous research also mentioned the fact that the COVID-19 pandemic had particularly affected mothers who had the greatest levels of worry regarding the risk of unemployment and reduced working hours during the pandemic [33]. Our findings also indicate that there is a potentially vulnerable population among parents who are 35 to 44 years of age that was more susceptible to high stress during the pandemic. This finding may appear surprising since it contradicts the results of previous studies that reported higher stress scores among younger people than among older ages [34]. A possible hypothesis that could explain this finding is that in light of the cancellation of youth sports and activity classes during the pandemic, parents with younger children might be less impacted in their daily routines by self-isolation and have less trouble with the new online educational environment. Another explanation might be that younger parents were more likely to have a single-child family and thus have to meet fewer child demands than parents with multiple children. Among sociodemographic factors, the present study did not find education to be a significant predictor of the level of perceived stress. The large percentage of highly educated participants in the sample might partially explain this finding. Another explanation might be that all parents, even those with a high level of education, might find it difficult to adjust to multiple roles in the family and at work because they might have to interrupt their work, rearrange their work hours, or receive less social support from family, colleagues and the community during this period in their day-to-day management of their children at home. Furthermore, vulnerability, environmental and social factors were revealed to make some parents more susceptible to high levels of stress. As expected and as previously demonstrated among different populations [2, 35], the occurrence of a COVID-19 diagnosed among acquaintances and/or family members predicted stress among parents. Traditional social support from extended family and friends was limited during the lockdown, and many parents lost access to common sources of leisure activity, such as community centers, churches, and fitness centers. Consistent with previous findings [36], high levels of support from family and friends were protective factors. While parents and children were completely reliant on each other during the lockdown, our findings revealed that parents with conflicts in the home and those who found it difficult to isolate themselves at home had significantly higher levels of stress. During quarantine, parents had to work from home while simultaneously engaging their children in homeschooling or virtual schooling activities, often without the possibility of occupying separate rooms [37]. A recent study also found that living alone or with few family members was a protective factor against perceived stress [32]. These results highlight that community organizations and social workers should pay more attention to the prevention of family conflicts and that applicable interventions might reduce conflicts and pressure. According to our results, a lower level of parental resilience and a worse mental self-perceived health status were important risk factors in our study**.** Our data are particularly relevant since the authors of recent studies have reported that the lowest levels of resilience were associated with higher levels of psychopathology [38]. As researchers have found that resilience is not stable but rather fluctuates as a function of life circumstance and type of intervention [39], our results underlined its potential key role in adaptating to COVID-19 as previously found for other pandemics and disasters [40, 41]. These results suggest that interventions to enhance resilience among parents might help to prevent or reduce the occurrence of stress in this population. Moreover, as prior research has shown that parental distress and parental mental health in disaster situations are associated with increased vulnerability to distress and poor mental health in children and adolescents [42] and that an improvement of the mental health on one member of the system can improve the mental health of the rest of the members of the system [38], our results highlight the need to detect vulnerable parents early, to build specific programs using existing resources to increase personal capabilities and to promote effective preventive programs to limit negative psychological outcomes for both parents and family. Developing coping strategies such as stress reappraisal, mindfulness-based stress reduction and the utilization of mobile phone mindfulness applications that could reduce parents’ distress has been suggested [43]. Health care providers and policymakers also need to provide solutions for these vulnerable groups with special consideration for their needs, including accessible mental health services.

Several limitations should be considered. First, the study was conducted via an online survey that required the use of the internet, including access to computers, smartphones or tablets. Thus, only internet users could be included. Second, there were no pre-COVID-19 assessments of parents’ mental health status, which warrants caution in interpreting the reported effects as consequences of the coronavirus disease. Third, another limitation of this study relates to its cross-sectional design, which prevents any assertion of causality and direction. Further studies using longitudinal designs are needed to confirm the hypothesized causal link between factors. Other limitations include the fact that our sample is not representative of fathers, since only 13.8% of the responders belonged to this category, which could overestimate the value of the odds ratio regarding the gender effect. Another limitation of the study is its inability to account for potential “school” effects in the multivariable regression model. Last, parental stress could have varied across geographical locations where different policies were in force at the time of survey completion. So, a larger sample size with individuals from different areas of France is needed to generalize the results. Despite these limitations, this study was the first French study to investigate the association between sociodemographic, environmental and psychological factors and the perceived stress of parents with children between the ages of 8 and 18 years old being under lockdown. It was conducted in a timely manner in the early stages of the COVID-19 epidemic and provides a starting point to develop specific psychological interventions aimed at decreasing stress in high-risk groups to deal with COVID-19 and other similar infectious diseases in the future.

## Conclusions

Our findings, which show that half of parents experienced moderate to severe stress, indicated that parents’ mental health should have been considered during the COVID-19 pandemic, which led to significant changes to almost all aspects of family living. The study identifies some personal, psychological and environmental variables that can aid health care professionals in the early detection of families at risk for maladjustment and can promote the utilization and development of necessary resources, targeted toward mental health management and particularly at mothers, to assist families. As the pandemic is ongoing and parental distress impacts mental health in children and adolescents, targeted interventions that consider either individual or relational levels need to be developed and applied to attenuate the negative health impacts of stressful situations and prevent the development of chronic outcomes.

## Data Availability

The data that support the findings of this study are available from the corresponding author upon reasonable request.
